# Socioeconomic inequalities in self-perceived oral health among adults in Chile

**DOI:** 10.1186/s12939-017-0519-9

**Published:** 2017-01-21

**Authors:** Francisco Gallego, Cristián Larroulet, Leonor Palomer, Andrea Repetto, Diego Verdugo

**Affiliations:** 1 Department of Economics, Pontificia Universidad Católica de Chile, Vicuña Mackenna 4860, Macul, Santiago, Chile; 2 Department of Philosophy, Logic and Scientific Method, London School of Economics and Political Science, Houghton Street, London, WC2A 2AE UK; 3 School of Dentistry, Pontificia Universidad Católica de Chile, Vicuña Mackenna 4860, Macul, Santiago, Chile; 4 School of Government, Universidad Adolfo Ibáñez, and Núcleo Milenio Modelos de Crisis (NS 130017), Diagonal Las Torres 2640, 234A Peñalolén, Santiago Chile; 5 Department of Economics, Brown University, 64 Waterman Street, Providence, RI 02912 USA

**Keywords:** Chile, Health-related quality of life, Socioeconomic gradients, Inequality, I14, I15, C21, C26

## Abstract

**Background:**

This paper studies the socioeconomic disparities in self-perceived oral health among Chilean adults and in the perceived physical, functional, psychological and social consequences of oral health.

**Methods:**

In February 2011, 1,413 residents of Metropolitan Area of Santiago, Chile, were interviewed using a standardized questionnaire and examined by dentists for dental status and oral health conditions. Only adults 18 to 60 years old affiliated with the public healthcare system were eligible to participate. We estimate socioeconomic gradients in self-perceived oral health and its distinct dimensions. We use the Heckman two-step procedure to control for selection bias given the non-random nature of the sample. In addition, we use a two-equation ordered response model given the discrete nature of the dependent variable.

**Results:**

There is a non-linear socioeconomic gradient in self-perceived oral health even after controlling for oral health status. The gradient is steep at the lower end of the income distribution and constant at mid-income levels. These socioeconomic disparities are also found for the psychological and social dimensions of self-perceived oral health, but not for the functional limitations and physical pain dimensions.

**Conclusions:**

The findings are consistent with inequities in the access to oral health services due to insufficient provision in the public sector and costly options in the private sector.

**Electronic supplementary material:**

The online version of this article (doi:10.1186/s12939-017-0519-9) contains supplementary material, which is available to authorized users.

## Background

Oral health disorders impact everyday life and the quality of life on multiple dimensions [1, 2]. Poor oral health is associated with functional limitations, pain and low self-esteem [3, 4], as well as with a number of general health conditions [5]. Other less studied dimensions such as labour market opportunities also relate to oral health [6].

Oral health-related quality of life depends crucially on the way individuals perceive oral conditions translating into their daily lives. For example, a poor oral condition might lead to eating difficulties and have a negative impact on nutritional intake [7, 8]; it might translate into functional limitations due to discomfort, restricting activities like work or study [2, 9]; and it might translate into reduced social interactions due to shame over missing teeth or chronic bad breath [10, 11].

This paper aims to study socioeconomic inequalities in the subjective assessment of oral health among adults in Chile. We emphasize the role of socioeconomic background since much of the observed variability has been related to factors like education and income [12]. Specifically, we examine the independent contribution of these socioeconomic factors in explaining self-perceived oral health (SPOH) after adjusting for oral health status and other determinants. We also study socioeconomic gradients in the distinct dimensions of SPOH: that is, in the perceived physical, functional, psychological and social consequences of oral health.

Most of the studies on oral health gradients have been conducted in developed countries where public healthcare is relatively strong and where the private sector plays a very important role in the provision and financing of dental care [13]. Even in these contexts the literature consistently finds a significant socioeconomic gradient. For instance, Sanders et al. [4], Locker [12], Wamala et al. [14] and Sabbah et al. [15] find steep gradients in oral health among adults in Australia, Canada, Sweden and the United States, respectively. Interestingly, in one of the few studies for developing countries, Gabardo et al. [16] find no association between oral health perceptions and household income among adults in Brazil.

The provision of public oral health services in Chile is quite limited, as it mainly offers primary services to pregnant women, 6-year-old children and 60-year-old adults who are covered by public health insurance, in addition to basic outpatient emergency services [17]. This limited provision of public services addressing complex problems is combined with an active private sector that offers costly services of heterogeneous quality. According to the study conducted by Vásquez, Paraje and Estay [18], the organization of the oral health care system in Chile has led to a markedly pro-rich inequity in dental visits.

Although the private sector also plays an important role in the provision of dental care in developed countries, the coverage of public insurance programs and of the public provision of oral health services tend to be much more extended in those countries than in Chile [19]. Consequently, differences in the structure of the dental healthcare system may lead to differences in the observed socioeconomic gradients.

Thus, this study has three objectives: first, to assess whether socioeconomic status accounts for differences in oral health perceptions in the adult Chilean population; second, to assess the shape of the gradient; and third, to examine the socioeconomic disparities in the physical, functional, psychological and social dimensions of SPOH.

## Data and methods

### Study population and sampling

In February 2011, 1,413 residents of the Greater Santiago area were enrolled in a program offering free dental care services, including dental prosthetics.^1^ The target population included all adults between the ages of 18 and 60 and affiliated with the public healthcare system. Enrolment was non-random as participants responded to advertisements published in mass media.

To participate in the program, individuals had to first apply by phone or by registering online. They were then asked to attend a meeting at the Pontificia Universidad Católica’s San Joaquín Campus in Santiago.

After obtaining informed consent, trained staff administered a questionnaire and qualified dentists conducted an extensive dental examination. The protocol was approved by the Human Ethics and Research Committee of the Pontificia Universidad Católica de Chile Economics Department.

### Study measures

The questionnaire collects information on demographic and socioeconomic characteristics (age, sex marital status, number and age of children, employment and education) as well as on oral health behaviour, such as frequency of daily tooth brushing and the time since the last dental appointment. The survey also collects information on self-perceived oral health and self-esteem.

To measure oral health-related quality of life, each participant answered the 14 questions of the Oral Health Impact Profile (OHIP-14) developed by Slade [20], translated into Spanish and validated by López and Baelum [21] in a Chilean population. The instrument collects information on seven dimensions: Functional Limitations, Physical Pain, Psychological Discomfort, Physical Disability, Psychological Disability, Social Disability, and Handicap. Questions are answered on a five-point Likert scale. Scores range between 0 and 56; a higher score indicates a worse perceived oral health-related quality of life.

To measure self-esteem, we use the Rosenberg Self-Esteem Test [22], validated for Chilean adults by Rojas-Barahona et al. [23]. The instrument consists in 10 statements on overall feelings of self-worth evaluated on a four-point Likert scale. Scores range between 10 and 40; a higher measure indicates a higher global self-esteem.

The dental examination that followed the interview was conducted on the basis of protocols designed by the World Health Organization [24]. Dentists collected information on the status of each tooth (healthy, caries, missing, etc.) as well as on other conditions such as periodontal disease, gingivitis and dental occlusion problems.

This dataset is complemented with data from the nationally representative 2011 CASEN survey, which collects rich information on the socioeconomic characteristics of Chilean households [25].

### Statistical analysis

We use regression methods to estimate socioeconomic gradients in perceived oral health; i.e., we estimate a model like

1 $$ {y}_1={x}_1{\beta}_1+{x}_2{\beta}_2+{u}_1, $$

where *y*_1_ is a measure of self-perceived oral health, *x*_1_ is a vector of socioeconomic variables, *x*_2_ is a vector of control variables and *u*_1_ is an error term. We are interested in estimating the parameters in vector β_1_. Standard OLS techniques may not be appropriate in this case for two reasons: (1) the discrete and ordinal nature of the dependent variable, and (2) the non-random nature of the sample.

When addressing the first problem, it is worth noting that our main dependent variable has 57 categories. A number of simulation studies have shown that when the number of ordered categories is relatively high (i.e., five or more), the dependent variable can be treated as continuous without significant losses in terms of the statistical properties of the estimates [26–28]. Therefore, linear models such as OLS provide accurate estimates in many situations. Even so, in addition to the linear model in Eq. (1), we also estimate an ordered multinomial response model [29]. The model assumes that there is an unobserved latent outcome variable y* [30]:

2 $$ {y}^{*}={x}_1{\gamma}_1+{x}_2{\gamma}_2+{u}_2=x\hbox{'}\gamma +{u}_2 $$

However, we do observe *y*_1_ such that

3 $$ {y}_1=j\  if\ {\mu}_{j-1}<{y}^{*}\le {\mu}_j $$

where *j* = 0, 1, 2, …, *J*, *μ*_0_ = − ∞ and *μ*_*J*_ = ∞. The unknown *μ* parameters are estimated along with the *γ* parameters, assuming *u*_2_ follows a standard normal distribution. That is,

4 $$ \Pr \left[{y}_1=j\right] = \Phi \left({\mu}_j-{x}^{\prime}\gamma \right)-\Phi \left({\mu}_{j-1}-{x}^{\prime}\gamma \right) $$

where Φ represents the standard normal cumulative distribution function. It is worth emphasizing that each γ coefficient represents the marginal effect of the corresponding regressor on the latent measure of self-rated oral health y*, and not on the observed outcome *y*_1_. Thus, the OLS estimates and the ordered response model coefficients are not directly comparable; i.e., the coefficients of the latter model must be interpreted with care. Another limitation of non-linear models is that the researcher needs to assume a specific distribution, an assumption that is not testable and that may not be accurate to the specific problem [31].

With all this in mind -- that is, because the choice between linear and ordered response models is not *a priori* clear -- below we report the estimation results for both the linear and the ordered response models. In addition, estimating both models allows us to analyse the robustness of our results to the empirical strategy given the models’ different underlying assumptions.

With respect to the non-random nature of the sample, a non-representative sample leads to biased estimates if sample selection is the result of unobservable characteristics that are associated with both participation and outcomes. Since individuals had to first apply and then travel to campus to participate in the study, it is plausible that SPOH and individual choice of whether or not to participate are correlated. We use a two-equation procedure to correct for potential sample selection bias. That is, we also estimate an equation that models participation in the sample:

5 $$ {y}_2=1\left[{x}_1{\delta}_1+{x}_3{\delta}_3+{v}_2>0\right] $$

where *y*_2_ is an indicator variable denoting whether an individual is observed (i.e., is in the baseline sample), *x*_3_ is a set of variables that affect the participation decision and *v*_2_ is an error term. We assume that *v*_2_ follows a normal distribution and estimate a probit model for this equation. To do so, we combine the baseline survey with a representative sample from the 2011 CASEN survey consisting in all individuals who comply with the eligibility criteria to participate in the study. To ensure identification, we include in *x*_3_ variables that are excluded from *x*_2_ –i.e., instrumental variables that are related to participation but which have no independent effect on self-perceived oral health. We use variables measuring the proximity to information sources on the program, in addition to the time–cost of traveling to the University campus, as instruments.

Estimating the participation equation using a probit model produces an estimate of the inverse of the Mills ratio (IMR). When the model for SPOH is linear, it can be shown that under certain assumptions the estimation of Eq. (1) including the IMR as a control variable yields parameter estimates that are consistent. Moreover, the statistical significance of the estimate of the IMR coefficient serves as a test for the null of no sample selection [30, 32].

When estimating the ordered response model, we assume that the errors *u*_2_ and *v*_2_ follow a bivariate normal distribution with correlation coefficient ρ. We use maximum likelihood estimation methods to jointly estimate the outcome and participation equations. A likelihood ratio test comparing the log likelihood of the joint model with the sum of the log likelihoods for the ordered response and participation models yields a test of the null hypothesis of no sample selection [33].

Finally, we use Chi-squared tests comparing the coefficients in vector β_1_ in the linear model and the coefficients in vector γ_1_ in the ordered response model to analyse the shape of the estimated gradients.

### Selected variables and definitions

Table 1 displays the variables selected for the model estimation. The dependent variable of the self-perceived oral health equation is the OHIP-14 score. Explanatory variables are classified into two main groups: socioeconomic and demographic characteristics, and clinically assessed oral health. The variables used as instruments for identification are also listed.

**Table 1:** Selected Variables

Variable	Definition
Dependent variable	
OHIP-14 score	Self-rated oral-health related quality of life (0 to 56; higher scores indicate a worse perceived oral health status)
Independent variables	
Socioeconomic and demographic characteristics	
Sex	Sex: male = 1 and female = 0
Age	Participant’s age in years at the time of the baseline survey
18–30	Age between 18 and 30 years: yes = 1 and no = 0
31–40	Age between 31 and 40 years: yes = 1 and no = 0
41–50	Age between 41 and 50 years: yes = 1 and no = 0
51–61	Age between 51 and 61 years: yes = 1 and no = 0
Head of household	Whether participant is head of his/her household: yes = 1 and no = 0
Married/partner	Marital status of participant (married or living with partner): yes = 1 and no = 0
Children under 5	Number of children under age 5 living in the household
Children aged 5–18	Number of children between ages 5 and 18 living in the household
Education	Highest educational level attained by participant
Middle or less	8th grade or less: yes = 1 and no = 0
Incomplete secondary school	Between 8th and 11th grade: yes = 1 and no = 0
Complete secondary school	12th grade: yes = 1 and no = 0
Higher Education	At least some higher education: yes = 1 and no = 0
Employed full time	Participant works full time: yes = 1 and no = 0
Employed part time	Participant works part time: yes = 1 and no = 0
Healthcare system	Health plan within the Public Insurance system
Public Insurance A (most vulnerable)	Public insurance plan A: yes = 1 and no = 0
Public Insurance B	Public insurance plan B: yes = 1 and no = 0
Public Insurance C	Public insurance plan C: yes = 1 and no = 0
Public Insurance D (least vulnerable)	Public insurance plan D: yes = 1 and no = 0
Public Insurance unknown category	Public insurance plan unknown: yes = 1 and no = 0
Clinically assessed oral health	
Any front missing teeth	At least one front missing tooth: yes = 1 and no = 0
Caries	Number of caries
Less than 6	Less than 6 caries: yes = 1 and no = 0
6–11	6 to 11 caries: yes = 1 and no = 0
12 or more	12 caries or more: yes = 1 and no =0
Teeth lost due to caries	Number of teeth lost because of dental caries
Less than 6	Less than 6 teeth lost: yes = 1 and no = 0
6–11	6 to 11 teeth lost: yes = 1 and no = 0
12 or more	12 teeth lost or more: yes = 1 and no =0
Malocclusion	Occlusion problems: yes = 1 and no = 0
Instrumental variables	
Distance to subway	Whether there is a subway station within a 2 km radius from the municipality’s population centroid where participant resides: yes = 1 and no = 0
Travel time to campus	Travel time in minutes from the municipality’s population centroid where the participant resides to the University Campus
Tertile 1	20 min or less: yes = 1 and no = 0
Tertile 2	Between 20 and 37 min: yes = 1 and no = 0
Tertile 3	37 min or more: yes = 1 and no = 0
Other variables of interest	
Oral health and behaviour	
Any missing teeth	At least one missing tooth: yes = 1 and no = 0
Any caries	At least one caries (treated or untreated): yes = 1 and no = 0
Prosthetic need	Clinically assessed prosthetic need (upper and/or lower): yes = 1 and no = 0
Reported daily tooth brushing frequency	Number of times a day the participant brushes teeth
Dental visit within past year	At least one dental care visit within the past 12 months: yes = 1 and no = 0
Bad or regular self-rated oral health	Bad or regular self-rated oral health: yes = 1 and no = 0
Rosenberg self-esteem scale	Global self-worth scale (10 to 40; higher values indicate higher global self-esteem)

Within the first group, we are mainly interested in measures of socioeconomic status. We use two sets of variables: educational attainment and the socioeconomic vulnerability categories defined by the Chilean public health insurance system [34, 35].^2^ In Chile, individuals make mandatory contributions to health insurance, choosing between the single public insurance (National Health Fund or Fondo Nacional de Salud, also known as FONASA) and several private health insurance companies (Health Insurance Institutions or Instituciones de Salud Previsional, also known as ISAPREs). FONASA is structured into four plans -- from plan A, which covers the most vulnerable individuals, to plan D for the least vulnerable -- depending on income and number of dependents. Plans also differ in co-payment and benefits. The services provided by the ISAPREs are relatively more expensive and only the highest income households take this option. According to the CASEN 2011 survey, 13% of the population is affiliated with an ISAPRE, and 85% of those affiliated with the private system belong to households in the top two income quintiles.

Other variables in this group include sex, age and marital status, children in the household and employment status.

Clinically assessed oral health impairments include missing front teeth, number of dental caries, missing teeth due to caries and occlusion problems.

### Descriptive statistics

Table 2 contains summary statistics for the survey.

**Table 2:** Descriptive statistics

Variable	Mean	SD	N
OHIP-14 score	33.29	12.05	1413
Socioeconomic and demographic characteristics			
Sex	0.28	0.45	1413
Age			
18–30	0.16	0.37	1413
31–40	0.24	0.43	1413
41–50	0.36	0.48	1413
51–61	0.25	0.43	1413
Head of household	0.70	0.46	1413
Married/partner	0.50	0.50	1394
Children under 5	0.21	0.47	1413
Children aged 5–18	0.91	1.03	1413
Education			
Middle or less	0.17	0.38	1394
Incomplete secondary school	0.19	0.39	1394
Complete secondary school	0.45	0.50	1394
Higher Education	0.20	0.40	1394
Employed full time	0.47	0.50	1413
Employed part time	0.20	0.40	1413
Healthcare system			
Public Insurance A (most vulnerable)	0.29	0.46	1413
Public Insurance B	0.30	0.46	1413
Public Insurance C	0.26	0.44	1413
Public Insurance D (least vulnerable)	0.14	0.34	1413
Public Insurance unknown category	0.01	0.12	1413
Clinically assessed oral health			
Any missing front teeth	0.35	0.48	1413
Caries			
Less than 6	0.84	0.37	1413
6–11	0.14	0.35	1413
12 or more	0.02	0.14	1413
Teeth lost due to caries			
Less than 6	0.44	0.50	1413
6–11	0.31	0.46	1413
12 or more	0.25	0.43	1413
Malocclusion	0.81	0.40	1413
Instrumental variables			
Distance to subway	0.50	0.50	1413
Travel time to campus			
Tertile 1	0.49	0.50	1413
Tertile 2	0.41	0.49	1413
Tertile 3	0.10	0.30	1413
Other variables of interest			
Oral health and behaviour			
Any missing teeth	0.93	0.26	1413
Any caries	0.98	0.14	1413
Prosthetic need	0.84	0.37	1413
Reported daily tooth brushing frequency	2.75	0.87	1413
Dental visit within past year	0.32	0.47	1413
Bad or regular self-rated oral health	0.98	0.14	1413
Rosenberg self-esteem scale	27.94	4.42	1413

*Abbreviation*: *SD* standard deviation, *N* number of observations

The sample is mainly comprised of female participants (72%) who have completed secondary education or higher (65%), and who are heads of households (70%). A large fraction of participants self-rate their oral health as poor or regular (98%), reporting an average OHIP-14 score of 33.29, consistent with the high prevalence of clinically assessed oral health problems. Almost all respondents have experienced dental caries (98%) and 92% have lost at least one tooth over their lifetimes. According to the Ministry of Health [36], the prevalence of caries in Chilean adults is 99% and the prevalence of tooth loss ranges between 80 and 99% depending on age. Despite the frequency of oral health impairments in the sample, only 32% of participants had visited a dentist during the past year.

Table 2 also reports a mean self-esteem indicator equal to 27.9, a level somewhat lower than the average of 32.5 reported by Rojas-Barahona et al. [23].

## Results

### Gradients in self-perceived oral health

We estimate socioeconomic gradients in SPOH using both the linear and the ordered response models. Table 3 presents our benchmark estimates of the SPOH determinants. Additional file 1: Table S1 in the appendix shows the estimation results for the participation equation.

**Table 3:** Self-perceived oral health regression results

	OLS		Heckman Two-step		Ordered Model		Ordered Model Sample Selection	
	Coef.	*p*-value	Coef.	*p*-value	Coef.	*p*-value	Coef.	*p*-value
Sex	−3.991	0.000	−2.433	0.042	−0.351	0.000	−0.092	0.411
Age (base: 18–30)								
31–40	3.912	0.001	2.807	0.029	0.338	0.000	0.144	0.197
41–50	5.970	0.000	4.622	0.001	0.515	0.000	0.265	0.032
51–61	7.219	0.000	6.429	0.000	0.637	0.000	0.447	0.000
Head of household	1.522	0.038	−1.506	0.456	0.161	0.008	−0.270	0.049
Married/partner	−0.364	0.609	−0.186	0.784	−0.030	0.636	−0.002	0.973
Children under 5	2.273	0.001	2.974	0.000	0.196	0.001	0.265	0.000
Children aged 5-18	0.253	0.327	0.498	0.161	0.016	0.497	0.047	0.053
Education (base: Middle or less)								
Incomplete secondary school	−1.184	0.217	−2.162	0.069	−0.113	0.182	−0.231	0.004
Complete secondary school	−3.455	0.001	−4.749	0.000	−0.320	0.001	−0.450	0.000
Higher Education	−6.031	0.000	−7.206	0.000	−0.536	0.000	−0.623	0.000
Employed full time	0.211	0.739	1.420	0.196	0.004	0.941	0.170	0.018
Employed part time	−1.018	0.251	−1.425	0.124	−0.116	0.130	−0.154	0.048
Healthcare system (base: Public Insurance A, most vulnerable)								
Public Insurance B	−2.493	0.002	−3.166	0.001	−0.231	0.000	−0.291	0.000
Public Insurance C	−2.164	0.039	−3.640	0.004	−0.209	0.021	−0.379	0.000
Public Insurance D (least vulnerable)	−2.382	0.006	−3.475	0.007	−0.223	0.003	−0.341	0.000
Public Insurance unknown category	−2.131	0.172	3.431	0.771	−0.262	0.053	0.518	0.204
Inverse Mills ratio (IMR)			−4.294	0.105				
Constant	32.623	0.000	42.148	0.000				
Observations	1,394		1,394		1,394		1,394	
R-squared	0.138							
LR test indep equations							6.04	0.014

*Abbreviations*: *OLS* Ordinary Least Squares, *Coef* estimated coefficient

The dependent variable is the OHIP-14 score. Independent variables are described in Table 1. The first two columns report OLS estimates without correction for sample selection. The next two correct for sample selection using Heckman’s two-step estimator. The third set estimates an ordered multinomial model, whereas the final set corrects the ordered multinomial model for sample selection. Clustered standard errors at the municipality of residence level are reported

Inv. Mills ratio (IMR) is the sample selection correction variable in the Heckman model. After estimating the participation equation (Additional file 1: Table S1), for each observation in the sample we compute $\hat{\lambda}$ =ϕ(x_3_ $\widehat{\beta_3}$)/Φ(x_3_ $\widehat{\beta_3}$), where ϕ is the density of the Normal Distribution and Φ is its cumulative

LR test indep equations (likelihood-ratio test) tests hypothesis that the errors of OHIP-14 and participation equations are uncorrelated in the ordered response model

The table’s first two sets of columns present the estimates of the linear model. The estimated IMR coefficient is marginally significant, indicating that the error terms in the SPOH and participation equations are weakly correlated. Therefore, the OLS estimates may be inconsistent due to selection bias, but given that the IMR is only marginally significant, it is not surprising that most of the OLS and Heckman two-step estimates are qualitatively similar. In other words, the bias may be relevant for some of the estimated parameters and not relevant for others.

The linear model estimations corrected for sample selection show a socioeconomic gradient in SPOH. The gradient is steep at the lowest socioeconomic category and relatively constant at higher levels. In fact, we cannot reject the hypothesis that the coefficients associated to the FONASA B to D variables are equal to each other (*p* = 0.88). The effect of ascending from FONASA A to a higher plan -- evaluated at the point estimate of the FONASA B coefficient -- is large and significant, equivalent to a drop of about 3.2 points in OHIP-14 scores (95% CI (−5.00,-1.34), and *p* = 0.001).

Educational attainment is also related to SPOH in the two-step linear model even after adjusting for material resources. Each additional educational category leads to statistically significant improvements in oral health-related quality of life. For instance, completing secondary school after having only completed middle school reduces SPOH by almost 2.2 points (95% CI (−4.49, 0.17), and *p* = 0.069).

The estimation results also suggest that self-perceived oral health is worse among women and that it is negatively correlated with age and with the number of children under 5 years of age in the household.

The last two sets of columns in Table 3 present the ordered response model estimates. It is worth emphasizing that the ordered multinomial response and linear coefficients are not directly comparable: the former measures the effects on the latent continuous self-rated oral health variable, whereas the latter measures the effects on OHIP-14 scores. Nonetheless, the two models yield similar conclusions. First of all, the log-likelihood ratio test is statistically significant, indicating that the error terms in the SPOH and participation equations are correlated, confirming the results of the Heckman estimates but with a more precise estimator.

Secondly, income is negatively associated with SPOH. Again, the estimated gradient is non-linear, with OHIP-14 scores that improve discretely when individuals overcome the lowest income level, but which are constant when higher levels are reached. That is, we cannot reject the hypothesis that the coefficients of the FONASA variables B to D are equal to each other (*p* = 0.61). The point estimate of the FONASA B coefficient indicates that the unobserved latent SPOH variable y* (Eq. (2)) declines by 0.29 points when the individual moves from plan A to plan B (95% CI (−0.41, −0.17); and p < 0.001).

Third, educational attainment is again significantly and negatively associated with OHIP-14 scores whose point estimates increase as higher education levels are reached.

Finally, female participants have worse self-perceived oral-health, as do those who live in households where there are children under five years of age. Furthermore, SPOH worsens with age.

Table 4 presents estimates of the determinants of SPOH including controls for clinical measures of oral health. The table shows that oral health impairments are important determinants of SPOH in both sample-selection corrected models. Malocclusion, missing front teeth, a large number of caries and missing teeth due to caries all worsen SPOH independently. In addition, while the age categories are statistically significant in the two-equation estimates in Table 3, they are no longer significant when oral health impairments are included as controls, suggesting that the observed correlation between SPOH and age is due to decaying oral health status over time.

**Table 4:** Self-perceived oral health regression results, controlling for oral health condition

	Heckman Two-step		Ordered Model Sample Selection	
	Coef.	*p*-value	Coef.	*p*-value
Sex	−2.977	0.009	−0.202	0.067
Age (base: 18–30)				
31–40	0.762	0.538	0.021	0.853
41–50	0.854	0.526	0.016	0.884
51–61	0.991	0.437	0.057	0.608
Head of household	−1.079	0.573	−0.170	0.248
Married/partner	−0.052	0.936	0.005	0.936
Children under 5	2.967	0.000	0.272	0.000
Children aged 5-18	0.559	0.097	0.050	0.031
Education (base: Middle or less)				
Incomplete high school	−0.730	0.519	−0.102	0.317
Complete high school	−2.650	0.022	−0.281	0.002
Higher Education	−4.237	0.001	−0.402	0.001
Employed full time	1.121	0.283	0.125	0.133
Employed part time	−0.973	0.267	−0.120	0.148
Public health system (base: Public Insurance A, most vulnerable)				
Public Insurance B	−2.539	0.004	−0.249	0.000
Public Insurance C	−2.960	0.014	−0.318	0.000
Public Insurance D	−1.930	0.115	−0.213	0.005
Public Insurance unknown category	5.534	0.620	0.583	0.097
Any frontal missing teeth	4.721	0.000	0.404	0.000
Caries (base: less than 6)				
6–11	1.784	0.036	0.165	0.005
12 or more	7.032	0.001	0.607	0.000
Teeth lost by caries				
6–11	3.445	0.000	0.284	0.000
12 or more	5.171	0.000	0.443	0.000
Malocclusion	1.291	0.078	0.121	0.055
Inverse Mills ratio (IMR)	−3.782	0.131		
Observations	1,394		1,394	
LR test indep equations			4.21	0.04

Regarding socioeconomic inequalities, results in Table 4 show that the income and educational gradients are attenuated once clinically assessed oral health status is controlled for. In the case of FONASA affiliation, we estimate relevant differences in SPOH between the most vulnerable group and the rest. Importantly, and analogous to our previous estimates, while individuals affiliated with FONASA plans B, C and D are significantly better off than those in plan A, there is no statistically significant difference between them (*p* = 0.59 and *p* = 0.44 in the Heckman and ordered response models, respectively).

In sum, our findings in Table 4 show that biological factors such as tooth loss are associated with SPOH. However, a central finding is that even after controlling for those biological factors, there is a non-linear socioeconomic gradient in SPOH, with OHIP-14 scores that improve discretely only when individuals overcome the lowest income level. We also find an educational gradient in SPOH, even after controlling for proxies for material resources and biological factors.

### Gradients in selected SPOH dimensions

Socioeconomic indicators could impact differently the distinct dimensions of SPOH. We now explore socioeconomic gradients in eight selected SPOH question scores. We focus on functional limitations and physical pain, in addition to psychological and social interaction impairments. We present the results in Table 5, including controls for oral health conditions as in Table 4, and present the estimates using the ordered response two-equation procedure.^3^

**Table 5:** Determinants of SPOH. Selected questions

	Functional limitations				Physical pain		Physical disability		Social disability				Psychological disability		Handicap	
	Sense of taste affected		Trouble pronouncing words		Painful ache		Interrupted meals		Irritable with others		Difficulties doing jobs		Embarrassment		Life less satisfying	
	Coef.	*p*-value	Coef.	*p*-value	Coef.	*p*-value	Coef.	*p*-value	Coef.	*p*-value	Coef.	*p*-value	Coef.	*p*-value	Coef.	*p*-value
Education (base: Middle or less)																
Incomplete secondary school	−0.012	0.904	0.033	0.810	0.049	0.604	−0.233	0.010	−0.088	0.454	−0.093	0.206	−0.027	0.825	−0.037	0.769
Complete secondary school	−0.040	0.714	−0.001	0.996	0.136	0.212	−0.386	0.000	−0.275	0.018	−0.233	0.003	−0.184	0.111	−0.271	0.009
Higher education	−0.159	0.139	−0.107	0.543	0.135	0.189	−0.396	0.002	−0.379	0.012	−0.324	0.001	−0.320	0.011	−0.435	0.001
Public health system																
(base: Public Insurance A, most vulnerable)																
Public Insurance B	0.007	0.928	−0.188	0.021	−0.130	0.111	−0.172	0.001	−0.201	0.002	−0.250	0.000	−0.142	0.047	−0.106	0.101
Public Insurance C	0.099	0.386	0.049	0.655	−0.051	0.640	−0.308	0.000	−0.240	0.021	−0.315	0.005	−0.238	0.035	−0.271	0.000
Public Insurance D	0.054	0.567	−0.012	0.926	−0.000	0.997	−0.164	0.028	−0.124	0.135	−0.234	0.036	−0.276	0.011	−0.123	0.207
Public Insurance unknown categ	−0.106	0.734	−6.045	0.000	−0.497	0.168	1.286	0.006	0.888	0.005	0.542	0.196	0.258	0.489	0.275	0.496
Children under 5	0.015	0.834	−0.032	0.603	0.062	0.329	0.281	0.000	0.197	0.004	0.217	0.000	0.227	0.002	0.245	0.000
Children aged 5–18	−0.032	0.276	−0.010	0.764	−0.021	0.338	0.057	0.053	0.044	0.099	0.072	0.005	0.068	0.002	0.052	0.093
Any frontal missing teeth	0.180	0.006	0.517	0.000	0.018	0.845	0.190	0.006	0.223	0.001	0.180	0.018	0.381	0.000	0.374	0.000
Caries (base: less than 6)																
6–11	0.170	0.072	0.090	0.319	0.162	0.035	0.071	0.152	0.061	0.338	0.067	0.287	0.213	0.008	0.114	0.088
12 or more	0.132	0.483	0.254	0.280	0.317	0.055	0.218	0.178	0.242	0.100	0.545	0.001	0.735	0.012	0.666	0.000
Teeth lost by caries																
6–11	0.035	0.564	0.188	0.021	0.022	0.738	0.174	0.028	0.160	0.079	0.174	0.004	0.385	0.000	0.240	0.000
12 or more	0.222	0.004	0.367	0.000	0.103	0.299	0.403	0.000	0.305	0.001	0.304	0.001	0.425	0.000	0.291	0.000
Malocclusion	−0.060	0.214	0.168	0.016	0.071	0.276	0.028	0.661	0.083	0.268	0.070	0.231	0.225	0.001	0.045	0.482
Observations	1,394		1,394		1,394		1,394		1,394		1,387		1,394		1,393	
LR test indep equations	2.94	0.086	1.41	0.235	2.71	0.100	11.44	0.001	2.04	0.153	3.55	0.059	2.31	0.128	5.64	0.018

*Abbreviations*: *Coef* estimated coefficient

We report the estimated coefficients for ordered multinomial models corrected for sample selection. The dependent variables are OHIP-14 subcategory scores. Regressions include all variables listed in Table 4; we report those related to socioeconomic status, children in the household and oral health status

We find no statistically significant correlation between FONASA categories, education or children at home and the functional limitations and physical pain scores. We do find steep income and educational gradients for all of the other selected questions. FONASA categories are non-linearly associated with these specific scores, with a threshold effect that separates the lowest public health insurance group from the remainder of the population. The number of small children in households is also a statistically significant determinant of these OHIP-14 dimensions.

Oral health impairment variables are also significant. Some have larger impacts on specific questions. For example, missing front teeth is an impairment that is very different from caries or malocclusion in terms of social significance and it has a larger impact on life satisfaction and on embarrassment.

In sum, after controlling for oral health indicators, our results imply that the psychological and social dimensions, rather than the physical limitation dimensions, seem to be responsible for the OHIP-14 gradients. Being embarrassed, irritable with others and feeling that life is less satisfying all show significant correlations with educational attainment and with FONASA categories. In all these cases, we again find that while individuals affiliated with FONASA B to D categories are significantly better off than those affiliated to FONASA A, their estimated coefficients are not statistically different between them (*p* = 0.46, *p* = 0.50, and *p* = 0.12, respectively).

## Discussion

This study investigated the association between self-perceived oral health and socioeconomic background among Chilean adults. Data comes from the baseline survey of a program offering free dental care services to low income individuals.

One relevant advantage of the survey is its rich information on clinically assessed oral health, self-perceived oral health, self-esteem, socioeconomic background and demographic variables. One limitation, however, is the exclusion of high-income households, precluding us from estimating the full socioeconomic gradient.

This sample, although not representative of the general population, should come close to the public policy target. That is, the estimation results should be representative of the target population of a policy to provide free dental care (i.e., adults between 18 and 60 years of age affiliated with the public healthcare system), as we corrected for self-selection. Moreover, we show that correcting for the self-selected nature of the sample does not seem to qualitatively affect the estimates of the effect of socioeconomic background on self-perceived oral health.

We found socioeconomic gradients in self-perceived oral health when using the healthcare insurance plan as a proxy for income. This estimated socioeconomic gradient is non-linear: while individuals at the lowest level report poorer self-perceived oral health conditions, individuals in all other categories are significantly better off but with no statistical difference between them. One caveat, though, is that the expectation of free dental care services may have led survey respondents to alter their assessment of SPOH. If this alteration of responses is correlated with income, the estimated gradient may be biased.

Although many studies have observed that individuals reporting worse oral-health related quality of life belong to low income households [12, 14, 15, 37–42], few studies have explicitly analysed the shape of the socioeconomic gradient [4].

Some studies assume a linear relationship between SPOH and household income in their multivariate analysis [12, 38, 40]. Other studies implicitly find a linear relationship between material resources and SPOH. For instance, Wamala et al. [14] measure material deprivation by combining several socioeconomic indicators, to find that the share of individuals self-rating their oral health as poor or very poor reduces approximately by half as the social deprivation index is reduced in a step-wise manner.

Sanders et al. [4] also find an approximately linear relationship between the likelihood of a fair or poor self-rated oral health when an index of relative social status is used as proxy for material resources. However, and in line with our results, the study finds a threshold effect characterized by a discrete deterioration in self-perceived oral health when household income falls below the second quintile of its distribution. Gabardo et al. [16] find a similar threshold effect for census tract average income, but no gradient for income measured at the household level.

We also found gradients associated with educational attainment even after controlling for material resources. Other studies have also found an independent effect of schooling on oral health [15, 37, 43], perhaps due to transitory income shocks that may be better captured by income variables and to health-related behavioural differences across educational groups.

The estimation results also show that SPOH is worse among women, and is negatively correlated to the presence of small children in the household. This probably occurs because of time- and money-consuming activities related to childcare, suggesting that individuals with small children could be an especially important target of oral health policies.

Finally, the analysis of specific SPOH scores suggests that the psychological and social dimensions, rather than the physical limitation dimensions, are responsible for the socioeconomic gradient in the OHIP-14 score. We find no statistically significant association between income measures or educational attainment and the functional limitations and physical pain scores. Trouble pronouncing words might be associated with specific missing teeth, a phenomenon that may not be correlated with income. In turn, the absence of a socioeconomic gradient for physical pain could be expected because tooth extraction is offered by the public system.

The analysis thus confirms the existence of a socioeconomic gradient in SPOH which we mainly attribute to inequalities in access to preventive dental services and to relatively complex oral health treatments like implants or prosthetics. Economically disadvantaged households face access barriers to dental healthcare in Chile given the limited public services provided and the costly and heterogeneous privately provided solutions. These barriers prevent even those with higher incomes within the public health insurance system to improve their SPOH.

The literature has linked socioeconomic status and health through channels other than the availability of material resources, in particular psychological factors like psychosocial threats that lead to stress and which are unequally distributed in society, as well as status-related patterns of health behaviour [44]. These factors could also influence our results, as they may operate simultaneously.

To determine whether psychological and psychosocial resources explain the relationship between socioeconomic status and oral health, some authors have included variables such as self-esteem in multivariate models of self-perceived oral health [12, 16, 37, 45, 46]. In general, they find that psychosocial variables have explanatory power in oral health regressions and that their inclusion reduces the strength of the socioeconomic gradient. These results have been interpreted as evidence favouring a role for psychosocial factors in explaining oral health disparities. However, other authors have suggested that self-esteem and life satisfaction can be explained by self-rated oral health; i.e., that causality may run in the opposite direction [47, 48].

In our sample, the simple correlation of the OHIP-14 and each of its questions with the Rosenberg measure of self-esteem is negative and strong, so better levels of SPOH are associated with higher self-esteem. However, as both OHIP-14 and self-esteem are outcome variables, important concerns arise regarding a regression analysis that treats one of these variables as exogenous to the other, preventing us from performing such a statistical analysis. Still, we cannot rule out the hypothesis that psychological factors play a role in explaining the gradients.

Similarly, it is plausible that oral health inequalities and behaviour impact each other [49, 50]. To avoid reverse causality problems, we have not included current behavioural variables in our models. In our sample there is a correlation between education and the frequency of dental care visits, but not between education and dental self-care behaviour such as frequent tooth brushing. If education serves partly as a proxy for long-term income, then this result is consistent with the hypothesis of unequal access to oral health services rather than with health-related behavioural differences. Sanders et al. [50] found a similar result.

We do control for clinical indicators of oral health that are partly determined by lifetime oral health behaviour. Socioeconomic gradients are attenuated after adjusting for oral status variables, although the socioeconomic background proxies remain significant and relevant predictors of self-perceived oral health.

Finally, there may also be concerns regarding whether the observed gradient is partly related to the effects of health on income as health affects the ability to work and to generate earnings. However, the kind of impairments we analyse in this study, such as tooth loss, are conditions that usually take a long time to reach their final stages [51]. Thus, if the causality runs from oral health status to income, then life course socioeconomic status, rather than present status should be identified as a main determinant of SPOH. We have included educational attainment as a measure of long term earnings potential. We find independent correlations between SPOH and this measure of lifetime earnings, and between SPOH and current income measures.

## Conclusions

This study contributes to the scant evidence on SPOH and socioeconomic status for developing countries. It also highlights the shape of this relationship in a context of unequal access to oral health services due to a combination of insufficient coverage in the public sector and costly and heterogeneous solutions in the private sector. Future research could theoretically and empirically examine the relationship between the organization of the oral healthcare system and the shape of the socioeconomic gradient.

This study analyses the case of Chile, a country that will probably make the transition to the group of developed countries in the next few decades. Thus, access inequalities could be even more pronounced in poorer countries and affect the shape of the socioeconomic gradient. For instance, access barriers could even extend to basic oral health services and not just to relatively complex procedures. If so, SPOH dimensions like those related to experiencing pain could show socioeconomic gradients, unlike in the Chilean case.

The appropriate policy interventions to reduce the observed inequities depend on the factors that explain the gradient. Although this study emphasizes inequity in access, the relevance of other channels that may link socioeconomic background to dental health should be examined more closely. Given the potential reverse causality problems, we cannot rule out the role these other channels play in explaining the variation in self-perceived oral health along the income distribution, in particular, of psychosocial factors. Future research could address these questions using experimental and quasi-experimental variation in access to dental care.^4^

## Additional file

Additional file 1: Supporting Information: Determinants of participation in the sample. **Table S1**. Probability of participating in the baseline survey sample. (DOCX 39 kb)

## Abbreviations

OLS: Ordinary least squares
SPOH: Self-perceived oral health

## Footnotes

1 Results of the program evaluation are in Gallego et al., in progress [52].

2 We use health insurance categories instead of income as a proxy for material resources because we suspect household income is underreported in our baseline sample, particularly for higher income individuals within the public health insurance system.

3 The linear model results yield similar conclusions and are available upon request.

4 For instance, Gallego et al. [52] analyse the impact of oral health on both SPOH and the Rosenberg scores in the context of an experimental design constructed using the sample of this study.
