# Supplementary material for: Socioeconomic inequalities in self-perceived oral health among adults in Chile
Source: Int J Equity Health. 2017 Jan 21;16:23. doi: 10.1186/s12939-017-0519-9 (PMC5251305; doi:10.1186/s12939-017-0519-9)
Supplement: Additional file 1: — Supporting Information: Determinants of participation in the sample. Table S1. Probability of participating in the baseline survey sample. (DOCX 39 kb) [file 12939_2017_519_MOESM1_ESM.docx]

**Supporting Information: Determinants of participation in the sample**

This appendix reports the determinants of the probability of participating in the sample (equation (5) in the main text). The dependent variable is a dummy variable that equals 1 if the individual applied to the program and 0 otherwise (i.e., individuals in the 2011 CASEN survey who comply with the program eligibility criteria). The independent variables are the demographic and socioeconomic variables listed in Table 1, as well as the variables selected as instruments. Table S1 reports the results of the probit analysis. The table shows that most of the covariates are statistically significant.

Participation is more likely among women and heads of household. Education and socioeconomic status (using FONASA plans as proxies) are also positively related to participation. In turn, participation is negatively correlated with the number of children in the household: each additional preschool age child reduces the likelihood of participation by about 2 percentage points, whereas each additional school age child reduces it by less than one percentage point. Full employment is also negatively correlated with participation. Possibly caring for small children and working full time reduce the likelihood of program awareness and application as they are time-consuming activities.

Variables excluded from the second stage (distance to the subway and travel time to campus) are significant and with the expected signs. The first variable indicates whether there is a subway station within a two-kilometre radius from the population centroid of the municipality where the individual lives. It is expected to capture location amenities and access to information about the program, since an important dissemination mechanism was a newspaper distributed for free in Santiago’s subway system. It may also capture other unobservable characteristics that may be correlated with residential location choices. In turn, travel time to campus, measured from the municipality’s population centroid, serves as a proxy for transportation costs. We assume travel during low-intensity traffic daytime since individuals could choose their preferred time to visit the campus.

We find that individuals who live in municipalities with close access to subway stations are nearly 2 percentage points more likely to participate in the sample. We also find that those in the third tertile of travel time to campus – who take 37 minutes or more – are 3.5 percentage points less likely to participate than those in the first tertile – who take less than 20 minutes.

**Table S1. Probability of participating in the baseline survey sample**

|  | Marginal Effect | |  |
| --- | --- | --- | --- |
|  | Coef. | p-value |  |
| Sex | -0.054 | 0.000 |  |
| Age (*base: 18-30*) |  |  |  |
| *31-40* | 0.044 | 0.000 |  |
| *41-50* | 0.054 | 0.000 |  |
| *51-61* | 0.027 | 0.000 |  |
| Head of household | 0.132 | 0.000 |  |
| Married/partner | -0.008 | 0.119 |  |
| Children under 5 years of age | -0.023 | 0.000 |  |
| Children aged 5-18 | -0.007 | 0.001 |  |
| Education (*base:* *middle or less*) |  |  |  |
| *Incomplete secondary school* | 0.033 | 0.000 |  |
| *Complete secondary school* | 0.043 | 0.000 |  |
| *Higher education* | 0.041 | 0.000 |  |
| Employed full time | -0.047 | 0.000 |  |
| Employed part time | 0.015 | 0.098 |  |
| Healthcare system (*base: Public Insurance A, most vulnerable*) |  |  |  |
| *Public Insurance B* | 0.023 | 0.000 |  |
| *Public Insurance C* | 0.063 | 0.000 |  |
| *Public Insurance D (least vulnerable)* | 0.046 | 0.000 |  |
| *Public Insurance unknown category* | -0.073 | 0.000 |  |
| Distance to subway | 0.019 | 0.062 |  |
| Travel time to campus (*base: Tertile 1*) |  |  |  |
| *Tertile 2* | -0.005 | 0.640 |  |
| *Tertile 3* | -0.035 | 0.011 |  |
|  |  |  |  |
| Observations | 13,006 |  |  |
| Pseudo R squared | 0.1801 |  |  |

Abbreviations: Coeff=coefficient.

The dependent variable is a dummy variable indicating whether the individual participated in the sample. We report the marginal effects of the probit model, using clustered standard errors at the municipality of residence level.
